# The Rise in Neurodevelopmental Referrals and Diagnoses in Children and Young People.

**DOI:** 10.1192/bjo.2026.11540

**Published:** 2026-06-30

**Authors:** Cerys Blenkiron

**Affiliations:** Cardiff University, Cardiff, United Kingdom

## Abstract

**Aims:**

Neurodevelopmental disorders, including ASD and ADHD, are lifelong conditions that typically present in childhood. Early identification and timely diagnosis are essential to facilitate access to appropriate support, reasonable adjustments and evidence-based interventions to improve long-term outcomes. In recent years, health services across Wales have reported a substantial increase in referrals for neurodevelopmental assessment. This trend has been observed in ABUHB, placing increasing pressure on neurodevelopmental diagnostic services and contributing to prolonged waiting times.

The purpose of this service evaluation is to examine patterns in neurodevelopmental referrals and diagnoses amongst young people within ABUHB, and to assess whether existing diagnostic pathways meet expected quality standards. The evaluation aims to describe referral volumes, waiting times and diagnostic outcomes for the young people referred for neurodevelopmental assessments. In addition, it seeks to identify trends over time in the volume and nature of referrals, including demographic characteristics, and to assess service performance against national and local guidance, including NICE recommendations and Welsh Government neurodevelopmental targets.

**Methods:**

To achieve these aims, a retrospective review of routinely collected service data from ABUHB’s neurodevelopmental pathway was undertaken. Quantitative analysis was used to evaluate referral rates, time from referral to assessment and diagnostic outcomes. Observational learning complemented the quantitative findings through attending neurodevelopmental assessment clinics and MDT meetings, providing insight into the clinical decision making, referral pathways and multidisciplinary working. Engaging with cliniciansinvolved in assessment further contextualised the findings and enabled deeper understanding of perceived challenges, service pressures and examples of good practice.

**Results:**

This service evaluation demonstrates increased demand for neurodevelopmental assessment, alongside extended wait times and variation in diagnostic outcomes across the pathway. Service data demonstrates a substantial rise in accepted referrals over time, rising from 1535 to 2221 between 2021 and 2023, with sustained high referral volumes after. These findings highlight areas where current practice aligns with recommended standards, and areas where service delivery may be constrained by capacity and resource limitations. The clinical observations provide valuable context regarding the impact of rising referral volumes on patient-centred care.

**Conclusion:**

In conclusion, this service evaluation will provide a structured, data-informed overview of the current performance and pressures within the neurodevelopmental diagnostic services within ABUHB. Through integrating quantitative service data with qualitative clinical observations, the project aims to generate evidence-based recommendations to support service improvement. This evaluation highlights the role of service evaluation in supporting reflective clinical practice and continuous quality improvement within child and adolescent mental health services.

